# An Isolated Left Common Carotid Artery Arising From the Ductus Arteriosus and a Complete Vascular Ring

**DOI:** 10.7759/cureus.87457

**Published:** 2025-07-07

**Authors:** Jaanak Patel, Ravi Ashwath

**Affiliations:** 1 Cardiology, Christus Health, Baylor College of Medicine, San Antonio, USA

**Keywords:** arch abnormality, carotid artery anomalies, isolated left common carotid, kommerell’s diverticulum, three-dimensional model

## Abstract

Arch vessel anatomy plays a critical role in both fetal development and postnatal cardiovascular function, with anomalies often linked to compressive symptoms or associated congenital defects. Isolated anomalies of the aortic arch, particularly those forming vascular rings, are rare and clinically significant due to their potential to cause tracheoesophageal compression. This article describes and underscores the rare and unique case of an isolated left common carotid artery (ILCCA), arising from the ductus arteriosus, with a complete vascular ring without intracardiac or genetic defects. The use of annotated 3D models significantly improved understanding of complex anatomy, emphasizing the anomaly’s physical basis, clinical relevance, and the importance of advanced imaging for diagnosis and surgical planning.

## Introduction

Isolation of the left common carotid artery is a rare anomaly with only 17 previously reported cases in several combinations [1-17]. In all cases, it was associated with a right aortic arch and an aberrant left subclavian artery, with the isolated left common carotid artery (ILCCA) arising from either the main pulmonary artery or the left pulmonary artery. The isolation is due to the vascular structure being disconnected from the aortic arch. Isolation of the left common carotid artery does not typically present as a vascular ring, which is a formation that encircles the trachea and esophagus, either partially or completely [1]. To the best of our knowledge, only one case has been documented describing an ILCCA with a complete vascular ring [2]. Our case falls into the second category, characterized by a complete vascular ring. We present a case of an ILCCA with a complete vascular ring in which we utilized annotated and segmented three-dimensional (3D) models to elucidate the complex anatomy.

## Case presentation

**Figure 1:**
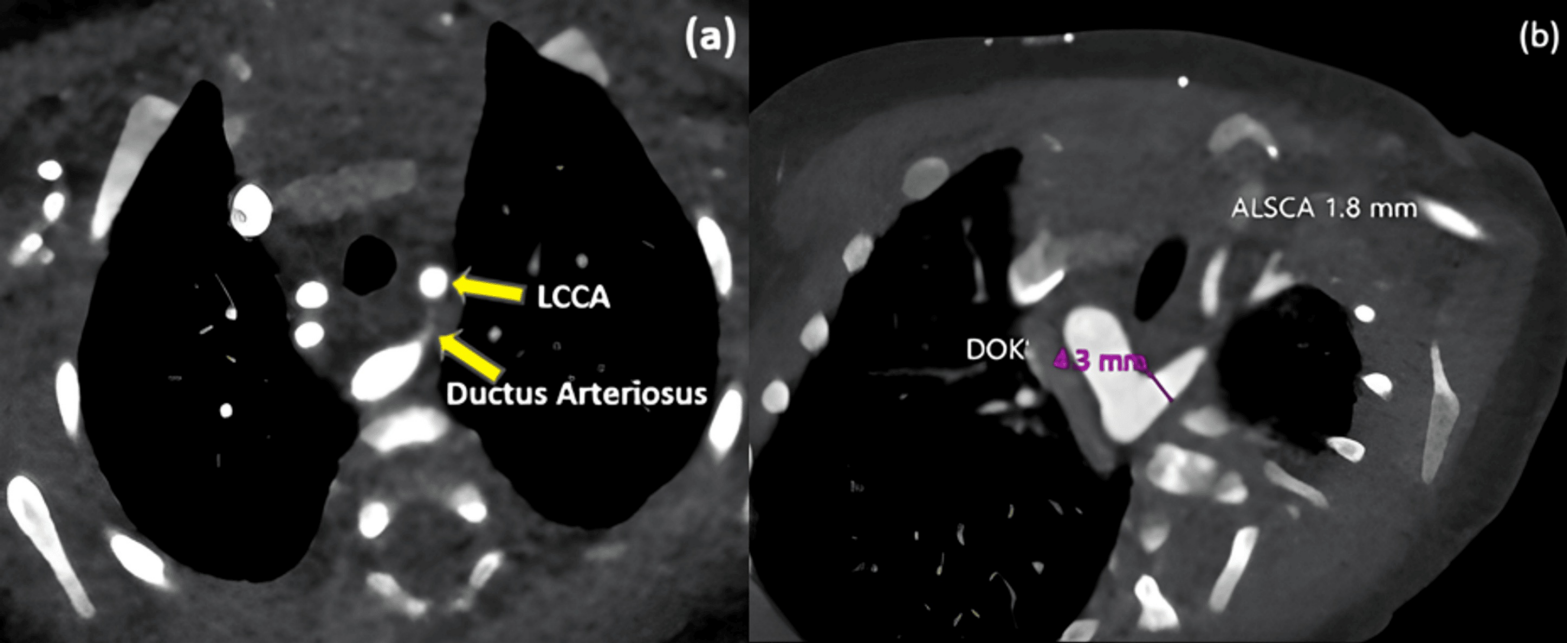
Contrast-enhanced CT depicting vascular anatomy (a) Axial Image showing LCCA with a probable origin from the ductus arteriosus. (b) Reconstructed image showing the right aortic arch, DOK, and an aberrant left subclavian artery. This configuration is consistent with a complete vascular ring. ALSCA: Aberrant left subclavian artery; DOK: Diverticulum of Kommerell; LCCA: Left common carotid artery

A term female infant was delivered vaginally following a pregnancy complicated by maternal iron deficiency anemia and pre-pregnancy BMI of 15. Prenatal ultrasound revealed bilateral renal pyelectasis and a suspected right aortic arch, prompting fetal echocardiography, which confirmed a right aortic arch with a left ductus arteriosus - findings consistent with a complete vascular ring. Prenatal genetic testing was declined. At birth, the infant weighed 2.77 kg, with Apgar scores of 9 at both one and five minutes. Routine post-natal echocardiogram on day 14 confirmed the presence of a right aortic arch with aberrant left subclavian artery and left patent ductus arteriosus forming a complete vascular ring, with no additional intracardiac defects aside from a patent foramen ovale. Due to the complexity of the anatomical arrangement, although asymptomatic, we obtained a computed tomographic (CT) on day 26 to better delineate and confirm the findings. A fast low-angle shot (FLASH)-gated CT angiogram of the chest was performed, and both 2D and 3D reconstructions were obtained. The images confirmed the anatomy noted in the echocardiogram, which included a right aortic arch with an aberrant subclavian artery (Figure 1a). Additionally, the isolated left common carotid artery was seen arising from the nearly occluded left-sided ductus arteriosus and a diverticulum of Kommerell (Figure 1b) with no signs of tracheal compression. The CT scan also showed trace contrast filling an almost closed ductus connecting to the LCCA. Due to the absence of a clear connection between the aorta and the LCCA, and with the ductus in the process of closing, we ordered a duplex ultrasound to assess whether the carotid artery had antegrade flow or was being filled retrogradely. This was done to evaluate for a potential steal phenomenon. Carotid duplex ultrasonography demonstrated antegrade flow in the isolated left common carotid artery.

Management

The infant remained clinically well with no signs of hypoperfusion or tracheal compression. Antegrade flow through the isolated left common carotid artery on duplex ultrasonography indicated adequate cerebral perfusion. Given the absence of symptoms and the surgical risks in infancy, a multidisciplinary team of cardiologists and surgeons recommended expectant management. Follow-up duplex at 9 and 18 months confirmed persistent antegrade flow without evidence of vertebrobasilar steal or retrograde filling via the circle of Willis.

## Discussion

An ILCCA is an exceptionally rare congenital vascular anomaly, with only 17 cases reported in the literature to date [1-17]. Among these, Juergensen et al. described the only known case of an ILCCA arising directly from the main pulmonary artery, independent of a patent ductus arteriosus (PDA), and associated with a complete vascular ring. That case required surgical reimplantation of the LCCA into the distal aortic arch, along with ligation and division of the left ligamentum arteriosum [2].

The embryologic origin of ILCCA remains a subject of debate. In early reports, including the first case described by Fong et al., the LCCA was connected to the pulmonary artery via a left-sided ductus arteriosus [3], supporting the theory of abnormal interruption of Edward’s hypothetical double aortic arch. However, subsequent cases have documented a left-sided ductus or ligamentum arteriosum anatomically distinct from the ILCCA [4], challenging this hypothesis. These findings have led some authors to propose malseptation of the truncoaortic sac as a more plausible embryologic mechanism [5]. In our case, the ILCCA appeared to arise from the left ductus near its junction with the pulmonary artery, aligning with this latter theory.

**Figure 2:**
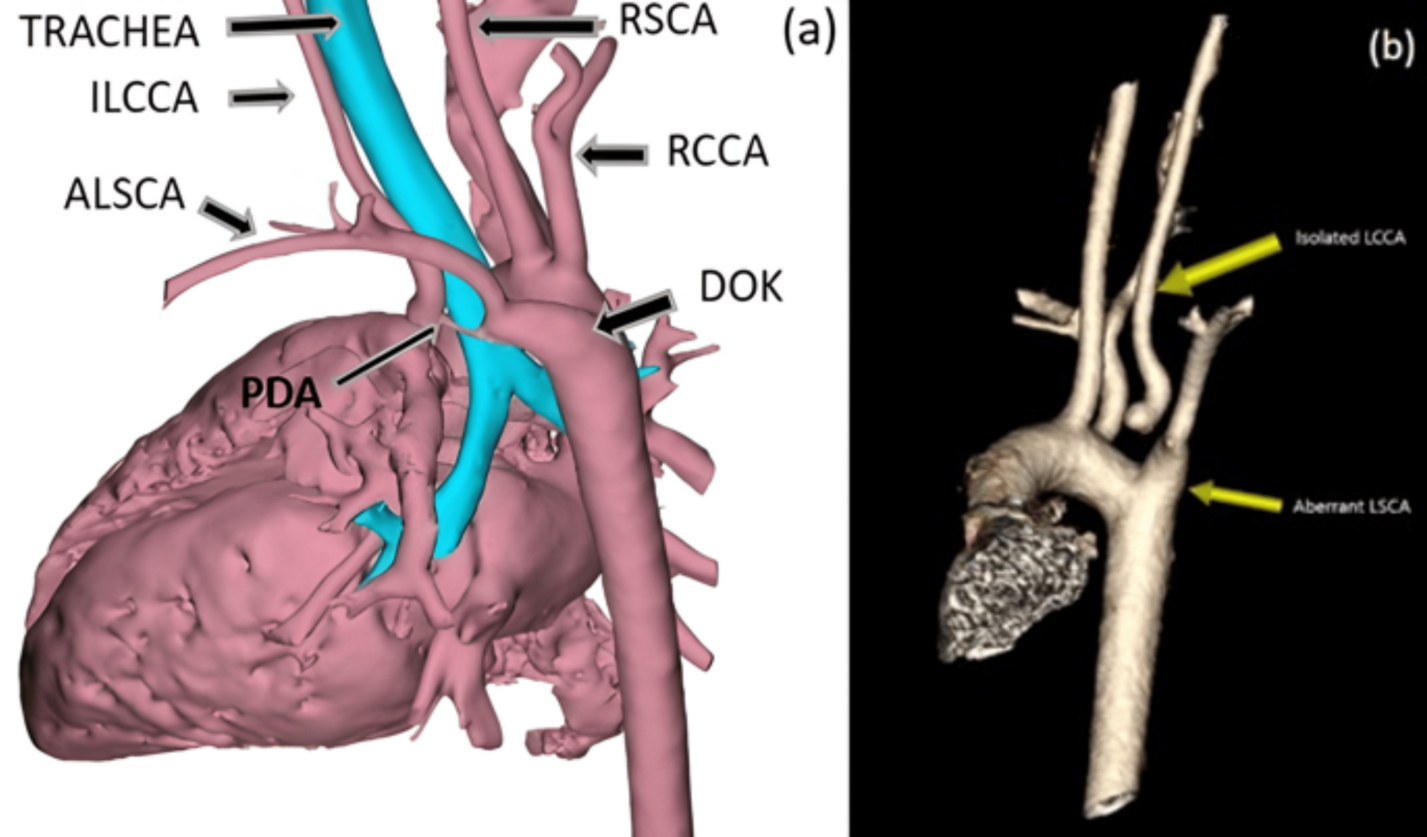
Annotated 3D models generated from the CT angiogram (a) 3D modelling showing the aortic arch branching and vascular ring circling the trachea. (b) 3D rendering of the aortic arch vessels showing the isolated LCCA and aberrant LSCA. ALSCA: Aberrant left subclavian artery; DOK: Diverticulum of Kommerell; ILCCA: Isolated left common carotid artery; PDA: Patent ductus arteriosus; RCCA: Right common carotid artery; RSCA: Right subclavian artery

Accurate diagnosis of such complex vascular anomalies relies heavily on cross-sectional imaging. Computed tomography (CT) angiography remains a cornerstone in delineating vascular anatomy, particularly in the context of congenital arch anomalies. In our case, CT imaging confirmed the presence of a right aortic arch with an aberrant left subclavian artery, a diverticulum of Kommerell, and an isolated LCCA arising from a nearly occluded left ductus arteriosus. To further enhance anatomical understanding, we utilized 3D modeling (Figure 2). The interactive 3D reconstruction clearly labels key structures - including the aortic arch, aberrant subclavian artery, diverticulum of Kommerell, and ILCCA - providing an intuitive and immersive visualization. This tool proved invaluable for our multidisciplinary team, facilitating both diagnosis and management planning for this rare and complex anomaly.

Follow-Up

At 15 months of age, the patient remains clinically well, with no symptoms attributable to the vascular ring - specifically, no evidence of tracheal compression or vertebrobasilar insufficiency related to the ILCCA. Serial carotid duplex ultrasounds performed at 32 days, 74 days, and nine months consistently demonstrated antegrade flow in both carotid arteries, indicating preserved cerebral perfusion. The most recent echocardiogram prior to the nine-month duplex did not visualize the PDA, suggesting possible spontaneous closure. At approximately nine months, concerns were raised regarding left-sided ptosis and clonus. Brain MRI revealed no evidence of infarction but did show hypoplasia of the distal cervical and intracranial segments of the left internal carotid artery, as well as a hypoplastic left A1 segment. Despite these findings, the child continues to thrive, and a conservative management approach has been adopted. Ongoing follow-up includes regular physical examinations, echocardiography, and carotid duplex imaging, with intervention reserved for the emergence of clinical symptoms or concerning imaging findings.

## Conclusions

An LCCA with a vascular ring represents an exceptionally rare vascular anomaly, particularly in the absence of intracardiac abnormalities and declined genetic testing. The presence of collateral circulation through the circle of Willis and the potential vertebrobasilar steal phenomenon underscores the need for close monitoring. Special attention should be given to evaluating for ductal tissue at the origin of the LCCA, as this may prevent complete isolation and impact management.

For symptomatic patients - whether due to the vascular ring or cerebral insufficiency - management strategies must be individualized, guided by clinical presentation and informed by multidisciplinary collaboration. Given the technical complexity and risks associated with surgical intervention on small-caliber vessels, careful preoperative planning is essential. Accurate diagnosis requires a high index of suspicion and a thorough imaging workup, including CT angiography, duplex ultrasonography, and long-term clinical surveillance. The integration of annotated and segmented 3D models offers a powerful tool for understanding complex vascular anatomy. They help improve spatial orientation and clarify relationships to adjacent structures, which is especially helpful for team members who may not be experts in cross-sectional imaging. These models also facilitate effective communication within care teams and with families, as they can enhance understanding and communication of complex vascular anatomy. As such, they are indispensable in the management of rare and intricate vascular anomalies such as an ILCCA.
